# Motivating HIV Providers in Vietnam to Learn: A Mixed-Methods Analysis of a Mobile Health Continuing Medical Education Intervention

**DOI:** 10.2196/12058

**Published:** 2019-04-18

**Authors:** Anna Larson Williams, Andrew Hawkins, Lora Sabin, Nafisa Halim, Bao Le Ngoc, Viet Ha Nguyen, Tam Nguyen, Rachael Bonawitz, Christopher Gill

**Affiliations:** 1 Department of Global Health Boston University School of Public Health Boston, MA United States; 2 Consulting, Researching on Community Development Hanoi Vietnam; 3 Center for Population Research Information and Databases General Office for Population and Family Planning Vietnam Ministry of Health Hanoi Vietnam

**Keywords:** continuing medical education, HIV/AIDS, mHealth, Vietnam, health education, SMS intervention, telemedicine, text messaging

## Abstract

**Background:**

The Mobile Continuing Medical Education Project (mCME V.2.0) was a randomized controlled trial designed to test the efficacy of a text messaging (short message service [SMS])–based distance learning program in Vietnam that included daily quiz questions, links to readings and online courses, and performance feedback. The trial resulted in significant increases in self-study behaviors and higher examination scores for intervention versus control participants.

**Objective:**

The objective of this mixed-methods study was to conduct qualitative and quantitative investigations to understand participants’ views of the intervention. We also developed an explanatory framework for future trial replication.

**Methods:**

At the endline examination, all intervention participants completed a survey on their perspectives of mCME and self-study behaviors. We convened focus group discussions to assess their experiences with the intervention and attitudes toward continuing medical education.

**Results:**

A total of 48 HIV specialists in the intervention group completed the endline survey, and 30 participated in the focus group discussions. Survey and focus group data suggested that most clinicians liked the daily quizzes, citing them as convenient mechanisms to convey information in a relevant manner. A total of 43 of the 48 (90%) participants reported that the daily quizzes provided motivation to study for continuing medical education purposes. Additionally, 83% (40/48) of intervention participants expressed that they were better prepared to care for patients with HIV in their communities, compared with 67% (32/48) at baseline. Participation in the online coursework component was low (only 32/48, 67% of intervention participants ever accessed the courses), but most of those who did participate thought the lectures were engaging (26/32, 81%) and relevant (29/32, 91%). Focus group discussions revealed that various factors influenced the clinicians’ decision to engage in higher learning, or “lateral learning,” including the participant’s availability to study, professional relevance of the topic area, and feedback. These variables serve as modifying factors that fit within an adapted version of the health belief model, which can explain behavior change in this context.

**Conclusions:**

Qualitative and quantitative endline data suggested that mCME V.2.0 was highly acceptable. Participant behaviors during the trial fit within the health belief model and can explain the intervention’s impact on improving self-study behaviors. The mCME platform is an evidence-based approach with the potential for adoption at a national scale as a method for promoting continuing medical education.

**Trial Registration:**

ClinicalTrials.gov NCT02381743; https://clinicaltrials.gov/ct2/show/NCT02381743

## Introduction

### Background

Continuing medical education (CME) is essential to maintaining the competence of a clinical workforce, but the complexity of managing CME programs and the resources required can be barriers to implementation. This may be particularly true in low- and middle-income countries. In November 2009, Vietnam passed the Law on Medical Examination and Treatment, which mandated that all clinicians participate in yearly CME activities to maintain licensure [1-3]. There has been increasing enthusiasm for mobile health (mHealth) in Vietnam, with 20 initiatives identified in a recent landscape analysis [4]. While this indicates an interest in using mHealth to improve the quality of health care through provider education, a lack of sustainability for current initiatives and absence of technological infrastructure pose challenges to mHealth programs across the country [4]. Since there is little infrastructure in the country to support such a national CME program, health officials may want to consider developing a feasible, scalable, and cost-effective platform that provides medical professionals with evidence-based training. Previous research in other contexts suggests that CME delivered at a distance is acceptable and can be effective at improving medical knowledge, changing behavior, and advancing clinical practice [5-15]. Distance learning is an attractive alternative to in-person CME, but evidence of its effectiveness is needed prior to program implementation at a national level.

Beginning in 2014, researchers at the Boston University School of Public Health, in Boston, MA, USA, collaborated with the Vietnamese Ministry of Health and the Vietnamese nongovernmental organization Consulting, Researching on Community Development to create the Mobile Continuing Medical Education Project (mCME). mCME was a short message service (SMS) text messaging–based mHealth education strategy that delivered CME via cell phones. Over 2 consecutive randomized controlled trials, we demonstrated that the mCME strategy was technically feasible, acceptable, and effective at motivating self-study behaviors, and it led to improved medical knowledge on a standardized examination [16-19].

Analysis of qualitative findings from the first mCME trial (V.1.0), which demonstrated feasibility and acceptability but not improved medical knowledge, influenced the design of V.2.0, which did demonstrate improved medical knowledge. During the design of V.2.0, mCME was considered a behavioral change intervention, and not a knowledge transfer intervention, with the emphasis on self-study behaviors. We enhanced the design to maximize study participation and engagement with the different intervention components. As we analyzed the results of mCME V.2.0, we noted that the fundamental components of the intervention were a pedagogical analog to the health belief model (HBM), a sociobehavioral theory that explains individual motivation to change behavior [20]. The HBM employs perceived susceptibility and perceived severity to rely on cues to action to encourage behavior change [20,21]. We posit that the intervention is aligned with the HBM because of the way that mCME spurred increased self-study, acquisition of evidence-based resources, and professional collaboration during its implementation.

### Objective

We aimed to analyze both qualitative and quantitative data obtained from participants randomly assigned to the mCME V.2.0 intervention to better understand the underlying mechanisms that led to successful increases in self-study behaviors and medical knowledge. We propose that understanding how the program worked will be critical to replicating its success at a national scale. We sought to understand how a digital education framework corresponding to the sociobehavioral HBM and designed to stimulate deeper learning could explain the success of the mCME approach. From within this framework, we used qualitative and quantitative data to evaluate (1) how medical professionals felt about the principal components in the intervention, and (2) the impacts that the intervention had on participants’ self-study, knowledge, and self-efficacy.

## Methods

### Study Site and Participants

Full details of the mCME V.2.0 project methodology are published in the main effects article [18]. Briefly, mCME V.2.0 was a randomized controlled trial conducted in 2016-2017 that aimed to test whether an integrative model of SMS text messaging and Web-based learning could improve medical knowledge among Vietnamese HIV clinicians. HIV health professionals from 3 provinces in northern Vietnam (Thái Nguyên, Hải Phòng, and Quảng Ninh) were enrolled in the study and took a baseline examination (1 held in each province) to assess medical knowledge and then randomly assigned into intervention and control groups. The intervention group received the following: a daily multiple-choice quiz question pertaining to a specific module within the Hanoi Medical University (HMU) online courses, daily linked readings to additional information, regular reminders to access the HMU online courses, and feedback on their individual performance versus their peers’ performance. The control group had access to the HMU courses and received nonmedical SMS text messages, but did not receive the daily medical quizzes, linked readings, feedback, or reminders to take the HMU courses. Multimedia Appendix 1 depicts the study design.

### Data Collection

At the end of 6 months, both intervention and control groups took an endline examination to test for improvement in medical knowledge and completed a survey on their study behaviors and experiences with the trial. The 50-question survey covered topics such as study behaviors, experiences with each of the intervention components, attitudes toward CME, job satisfaction, and perception of HIV knowledge and skills. After the endline examination workshops at each of the 3 sites, study investigators in Vietnam scanned the quantitative surveys with an optical scanning device (Scantron, Inc, Eagan, MN, USA).

After the endline examination, we invited a subset of intervention participants from each of the 3 provinces to participate in focus group discussions (FGDs) to learn about their experiences with the intervention. Within each group, we attempted to include a balanced representation of experiences with the intervention, with roughly equal numbers of participants whose response rates of the daily quiz questions were above the median, and those whose response rates were below the median. We used quiz response rates for this stratification because the daily quizzes were the most fundamental core element of the mCME intervention. Following a semistructured FGD guide, we asked participants about their experiences with the intervention, the impact the intervention had on their learning, and their suggestions for how to improve the mCME approach. All 3 FGDs were recorded in Vietnamese and translated and transcribed into English.

### Data Analysis

Boston University researchers analyzed the FGD transcripts in NVivo version 11 (QSR International). Themes and subthemes were individually identified and cross-checked by 2 qualitative analysts to test for variability, and then a consensus of key themes was reached prior to analysis. Responses were grouped and prioritized by frequency. We also compared responses by study site. Boston University researchers analyzed the survey data with various descriptive data analysis techniques using SAS version 9.4 (SAS Institute). As a secondary analysis, we analyzed qualitative and quantitative responses based on use of the intervention components (eg, we included survey results data on perspectives on the HMU courses only if the participant ever accessed the HMU courses). We include those results in this paper, to account for social desirability bias.

### Developing the Theoretical Framework to Understand Views on and Impact of the Intervention

**Figure 1:**
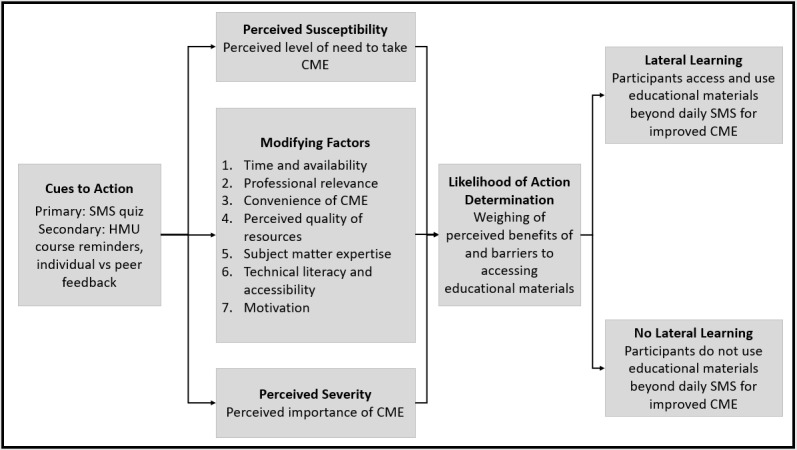
Framework for the decision to engage in lateral learning. Elements of the Mobile Continuing Medical Education Project are analogous to the health belief model. The figure illustrates the underlying behavioral mechanisms that lead a clinician to decide whether to engage in lateral learning, defined as accessing and using high-quality educational materials beyond daily short message service (SMS) text messages. CME: continuing medical education; HMU: Hanoi Medical University.

In a preliminary review of the data, we realized that there were several themes from the qualitative data that paralleled the HBM. The first component of both the HBM and our intervention is cues to action, which the HBM states provide a stimulus to trigger a decision or behavior change [20,21]. Another way in which this intervention mirrors HBM is through the idea of perceived severity and susceptibility; the HBM defines these as the individual’s own perceptions of the likelihood of getting a disease or having a severe form of the disease [20,21]. We have applied this concept beyond the context of disease; in this intervention, perceived severity and susceptibility correspond to the perceived importance and level of need to take CME to provide high-quality medical care. In our intervention, these views were informed by several modifying variables that affected quality and intensity of learning among the participants. We analyzed how participants chose to access and use high-quality educational materials beyond the daily SMS text messages, which we termed lateral learning. We then developed a framework to explain the clinician’s decision to learn through a behavioral lens (Figure 1 [20,21]). We analyzed the qualitative data within the context of this framework to explore how clinicians interacted with the intervention and to understand how lateral learning could be achieved.

The cues to action in mCME are the various text message interactions that the user has with the mCME program: the daily SMS quizzes, which prompt the user to seek an answer to a medical question; the HMU course reminders, which come at the beginning and end of each module; and individual and peer feedback, which comes in the form of a correct versus incorrect response per daily quiz question and an end-of-module final score comparing the individual’s percentage correct responses compared with the rest of the cohort. Whether the cues to action led to lateral learning depended on the individual’s weighing of the perceived susceptibility and perceived severity, which are modified by factors such as professional relevance, convenience of CME, perceived quality of resources, subject matter expertise, motivation and self-efficacy, technical literacy, and the participant’s time and availability. These factors should be considered together with the HBM’s concept of perceived susceptibility, or in this case, the perceived level of need for the individual to have CME, and the perceived severity, or in this context, the level of importance the individual places on CME. The individual then weighs the perceived prohibitive and supportive factors and determines whether to engage in lateral learning. Because a participant might choose to access vetted materials or additional resources of varying quality, we hypothesize that the educational value of outside resources is an additional moderating variable for lateral learning.

### Ethical Review

This study was registered on ClinicalTrials.gov (NCT02381743) with ethical oversight by Boston University Medical Center, Boston, MA, USA, and Hanoi University of Public Health, Hanoi, Vietnam. All participants in this trial provided written informed consent prior to participation in the trial, with additional consent provided for participation in the FGDs.

## Results

### Background Characteristics of mCME V.2.0 Trial Participants

Endline examinations with postintervention surveys and FGDs took place in May 2017. All HIV clinicians who participated in the intervention and returned for the endline examination were asked to complete the survey (n=48). We asked 10 clinicians assigned to the intervention group at each of the 3 sites to participate in the FGDs (n=30), all of whom signed a separate consent form specific to the qualitative research. Here, the term “intervention participants” includes all intervention participants, and “FGD participants” includes only the views of intervention participants who also participated in a focus group. Each FGD lasted 1 to 1.5 hours. The average age of all intervention participants was 41 years, and they had spent an average of 4 years in the HIV/AIDS field. As Table 1 shows, the demographic characteristics of the FGD subset and the full intervention cohort were generally similar, suggesting that the FGD subset was broadly representative of the larger group.

Below we first explore the various themes from the survey and the FGDs, including participant attitudes toward the major components of the intervention. We then describe the impact on self-study in relation to the modifying factors that influence learning behaviors, one of the key components to the lateral learning framework. Finally, we analyze the impact the intervention had on knowledge and self-efficacy.

### Views of the Intervention

**Figure 2:**
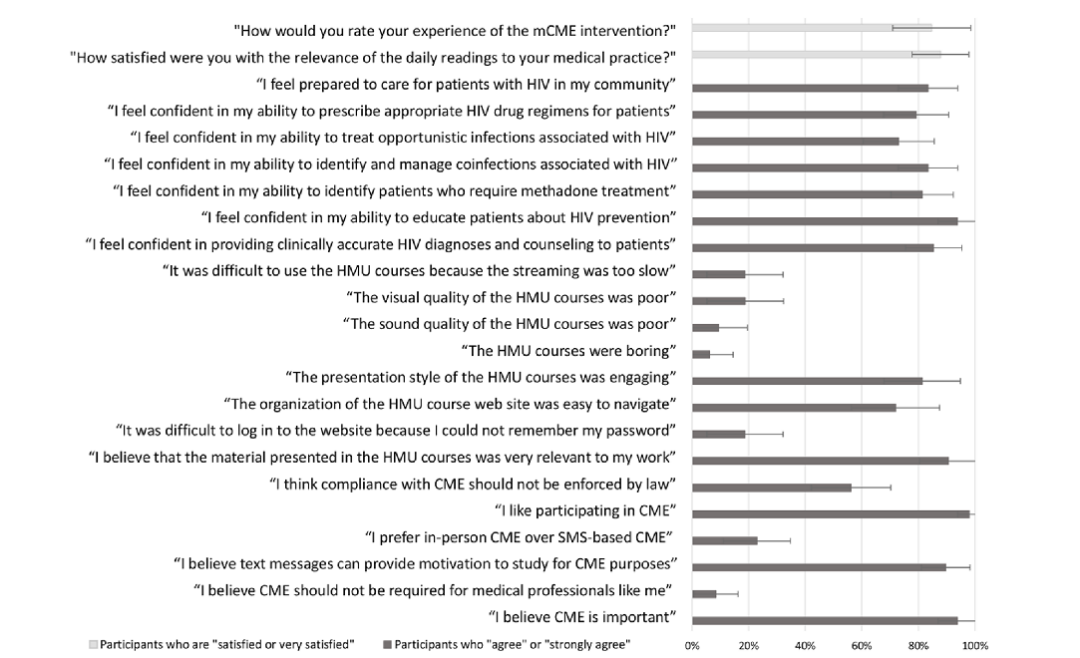
Sample of Likert-scale responses from Mobile Continuing Medical Education Project (mCME V.2.0) trial intervention participants in the endline survey showing the proportion of those who agreed or strongly agreed with the statements shown (standard error bars with 95% CI). Importantly, for questions pertaining to the daily readings and the Hanoi Medical University (HMU) courses, we excluded the responses from participants who never accessed those intervention components. CME: continuing medical education; SMS: short message service.

Similar to the results of mCME V.1.0, participants reported positive views of the intervention and of CME in general for mCME V.2.0 [17]. Of the 48 intervention participants, 45 (94%) agreed or strongly agreed with the statement “I believe CME is important” and 43 (90%) agreed or strongly agreed that text messages can provide motivation to study for CME accreditation (Figure 2). Additionally, 41 of the 48 (85%) intervention participants who accessed all 3 components of the intervention rated their experience as “very satisfying” or “somewhat satisfying.”

Intervention participants commented on their experiences with the 3 main components of the intervention: the daily quizzes, the daily readings that corresponded to the quiz question, and the HMU courses. Figure 2 shows additional survey data indicating the percentage of participants who agreed or strongly agreed with Likert-scale questions from the endline survey.

**Table 1:** Demographic characteristics and intervention behaviors of Mobile Continuing Medical Education Project (mCME V.2.0) trial participants.

Characteristics			Intervention participants (n=48)	Focus group participants (n=30)
**Sex, n (%)**				
	Male		19 (40)	11 (37)
	Female		29 (60)	19 (63)
**Research site, n (%)**				
	Thái Nguyên		18 (38)	10 (33)
	Hải Phòng		17 (35)	10 (33)
	Quảng Ninh		13 (27)	10 (33)
Age (years), mean (SD)			41.1 (8.8)	40.43 (8.7)
Years working in HIV/AIDS health sector, mean (SD)			4.3 (4.8)	5.20 (5.2)
**Clinical degree, n (%)**				
	MD		20 (42)	13 (43)
	Mid-level provider		28 (58)	17 (57)
**Text message response rate^a^ during study, by location, n (%)**				
	**Thái Nguyên**			
		High	13 (72)	5 (50)
		Low	5 (28)	5 (50)
	**Hải Phòng**			
		High	11 (65)	5 (50)
		Low	6 (35)	5 (50)
	**Quảng Ninh**			
		High	10 (77)	7 (70)
		Low	3 (23)	3 (30)
**Hanoi Medical University course use during study, n (%)**				
	Ever		32 (67)	19 (63)
	Never		16 (33)	11 (37)
**Daily readings access during study, n (%)**				
	Ever		41 (85)	22 (73)
	Never		7 (15)	8 (27)
**Hours per week spent on medical self-education, n (%)**				
	0		1 (2)	1 (3)
	1-2		26 (54)	17 (57)
	2-4		9 (19)	6 (20)
	4-7		7 (15)	3 (10)
	≥8		5 (10)	3 (10)
**Number of patients seen per day on average, n (%)**				
	0-9		23 (48)	15 (50)
	10-19		7 (15)	5 (17)
	20-29		8 (17)	4 (13)
	30-39		4 (8)	2 (7)
	≥40		6 (12)	4 (13)

^a^For response rates, high refers to those ≥85% for Thái Nguyên, ≥82% for Hải Phòng, and ≥77% for Quảng Ninh.

#### Daily Quizzes

Most FGD participants liked the daily quizzes, finding them to be convenient, relevant, and informative. This is consistent with daily quiz response rates during the intervention, which averaged 81.9% (118/144 daily quizzes) across 3 sites [19]. A few participants even wished that the intervention would continue; one commented that, “I quite miss the daily quizzes now that the intervention is over” (participant in Hải Phòng) and another said, “There have been no quizzes in the last 2 weeks and I miss them” (participant in Quảng Ninh). Remarking on the convenience of the daily quizzes, one participant said, “I feel satisfied because it is clear whether I have answered right or wrong” (participant in Quảng Ninh). Several participants reported that the daily quizzes led them to access educational materials to learn more about the topic area; 1 participant noted that they “encourage us to search for knowledge to be able to answer the questions—we had to read books or access online materials” (participant in Thái Nguyên).

Although most liked the quizzes, FGD participants commented that the two main drawbacks of the daily quiz were the formatting and system errors. Referring to phonetic markings and clarity, one person said, “I think the contents of the messages were not clear: choice a and b were sometimes put together in one line with no punctuation” (participant in Thái Nguyên). All participants in the FGD in Quảng Ninh agreed that the texts on the phone were too short, which affected their ability to understand what was being asked. Finally, participants at all 3 sites mentioned that there were sometimes system errors with the messages. One participant said, “Sometimes I chose the right answer but the system said I was wrong” (participant in Hải Phòng) and another said, “Some questions are vague, for example a question has many [correct] answers” (participant in Quảng Ninh). In this particular case, there was indeed one instance in which the programmers listed the wrong answer for a daily quiz; however, the claim that some quizzes had multiple correct answers was untrue.

Notably, most participants suggested that texts be sent in the morning and 24 hours be allotted to answer each SMS text message. One participant said that:

The timing of the messages in the afternoon is not reasonable. I had no time to search for more knowledge, since we were often busy in the afternoon at work, and at home we were too tired to research more.

participant in Thái Nguyên

We intentionally changed the timing of when the daily quiz was sent each day about halfway through the trial to test the impact of timing on the response rate. Most participants agreed that the timing of the messages (1:00 PM) negatively affected their ability to respond or seek out answers from provided resources. Importantly, although participants strongly protested against afternoon daily messages as opposed to morning messages, the response rate and correct answer rate was not significantly different between the 2 time points (Multimedia Appendix 2).

#### Daily Readings

Of the 3 intervention components, the daily readings had the lowest rates of utilization. The intervention participants accessed, on average, 18.1% (26/144) of the daily readings that they had received over the course of the intervention [19]. Many explained that the daily readings didn’t have enough in-depth information to cover complex topics. One participant elaborated:

I also think the contents in the links were not enough...when I accessed the links I didn’t understand completely. I still had questions and had to find other resources. If one doesn’t have enough knowledge on HIV then it would be difficult to understand contents of the link only.

participant in Thái Nguyên

For some, the length of the readings was sufficient and time efficient, but other participants sought more in-depth knowledge to gain deeper insight into the topic area.

Some participants found the daily readings to be a quick way to access information related to the daily quizzes. One participant noted that the daily readings in concert with the daily quizzes improved understanding:

Receiving daily messages probably helps me a lot, since I’ve just started [providing] ARV treatment for 3 months. It helps me in reading materials and providing information, thus enabling my self-study.

participant in Thái Nguyên

Another said that:

The links to the daily readings are great because they provide immediate answers.

participant in Quảng Ninh

Of the participants who accessed the daily readings, 88% (35/41) said that they were satisfied or very satisfied with the relevance of the daily readings to their medical practice (Figure 2). Despite these noted benefits, many participants complained that the daily readings were insufficient to improving their medical knowledge. One participant said simply that:

The contents of the daily readings are insufficient, especially for case questions.

participant in Quảng Ninh

#### Hanoi Medical University Courses

For the primary end point of this study, we observed that the intervention participants accounted for 83.2% of the total course use across intervention and control groups (134/161 total times accessed by all users), and intervention participants were significantly more likely than the control participants to ever access the HMU courses (relative risk 2.3, 95% CI 1.4-3.8) [18]. Few participants regularly accessed the HMU courses, but most of the intervention participants who ever accessed the courses thought the lectures were engaging (26/32, 81%) and relevant (29/32, 91%). One participant commented that:

If it was a topic I’m interested in then I’d watch it carefully. Some contents were very in-depth...[and the] assessment was very interesting, since it allowed me to see how much I could do, if I am progressing or not.

participant in Thái Nguyên

Most approved of the quality of the courses in terms of content, sound, visual elements, and navigation, according to the endline survey (Figure 2).

Reasons for not using the HMU courses included the participant’s lack of availability, topic relevance, and motivation. Speaking to why someone wouldn’t take the course, one person noted that:

Some knowledge I have already owned and have been directly trained, which makes it boring although I really like studying....Also, for the same lectures, reading documents only takes me 15-20 minutes, while listening to lecturer is too time consuming and I am sometimes busy, although it helps me gain more knowledge.

participant in Quảng Ninh

Another noted availability and course quality as deterrents:

I rarely use the HMU courses because the lectures are lengthy and unattractive despite being useful. First, they are just speeches or slides. Second, although they are beneficial, sometimes I am busy for 1, 2 days or even a week so I may forget to answer and there is no reminder from the system.

participant in Quảng Ninh

Many also suggested in the FGDs that the HMU lectures be updated to reflect current treatment guidelines; as one participant commented:

I hoped the knowledge would be updated according to the current treatment knowledge. At least we should be able to know the current possibility in treatment so we could give suitable consultation for patients.

participant in Thái Nguyên

Accessing the lectures on their mobile phones was also challenging. One person said, “The speed of loading links is quite slow for phones with low configuration, making it difficult to access” (participant in Quảng Ninh) and another noted that, “The online training provided by HMU loaded slowly on mobile phones despite a good Wi-Fi connection” (participant in Thái Nguyên).

Finally, several noted the limitations of mCME versus traditional CME when discussing the online courses. Many participants agreed that e-learning is the most convenient method. One participant said that:

This approach is very convenient, aiming to the majority of learners, any type of individual and convenient for community-based health staff.

participant in Thái Nguyên

However, several participants expressed more ambivalence toward online training:

This method does not have interaction between teachers and students so I feel unsatisfied because I cannot ask questions. But this method enables me to choose whichever part I like to study.

participant in Quảng Ninh

Others suggested that direct training be combined with mCME:

[With mCME], we get the convenience and can get access to it anytime, but especially in the treatment aspect, direct training with feedback would be more efficient and help keep a longer memory. Therefore, I suggest that online learning and direct training should be combined.

participant in Thái Nguyên

### Impact on Self-Study: Modifying Factors That Influenced Learning Behaviors

Our study results indicate that, compared with pretrial surveys, participants in the intervention group at endline reported higher levels of self-study through reference to medical textbooks, colleagues, online research, medical specialty websites, Vietnamese treatment guidelines, and scientific literature than their control group counterparts [18]. In the focus groups, participants raised several factors that influenced their decision to engage in self-study beyond memorizing the answer from the daily multiple-choice quiz. Among these factors were their time or availability, relevance of the subject matter to their profession, convenience of the intervention, perceived quality of the HMU and daily readings resources, individual-level subject matter expertise, technological literacy, and motivation to learn (Table 2).

Each of these factors fits within the lateral learning framework outlined in Figure 1. Table 2 provides exemplar quotes detailing the various factors that influenced participants’ decisions to engage in this lateral learning, or learning beyond rote memorization of the SMS text message.

Participant responses to the cues to action (SMS text message quizzes, course reminders, and performance feedback) were contextual, and the modifying factors listed in Figure 1 either aided in self-study or inhibited it. For example, participants studied more if they found the material to be professionally relevant, and did not study if they found the inverse.

**Table 2:** Quote excerpts from focus group discussions illustrating modifying factors in the decision to engage in lateral learning, May 2017.

Factor	Quote	Participant location
Time or availability	Honestly I don’t have much time so I would not access [additional materials].	Thái Nguyên
	*Q: Is there anybody who answered without checking any source?* All: Yes, when we are too busy.	Hải Phòng
	There are other things to do apart from studying. Actually, there are very few people who have time to participate in the courses at home, and studying when working often gets interrupted.	Quảng Ninh
Professional relevance	I would spend more time studying work-related topics, I researched more and often got a high score; other than that I would skip.	Thái Nguyên
	The [materials] are directly related to my work, so I need to study to improve my knowledge.	Thái Nguyên
	I believe that people directly treating patients would know more than us, so for those who do not know much about HIV/AIDS like us, reading is very important.	Thái Nguyên
Convenience	I could study at home. There is no need to go to class, so it is time and cost effective.	Hải Phòng
	All: Studying on our phones is more convenient because it is not possible to bring our laptops along all of the time.	Quảng Ninh
	The most beneficial thing for us working at medical centers is that we can study anywhere, without having to attend classes; it is suitable for those who live far away from the training centers.	Thái Nguyên
Perceived quality of resources	The links provide more information from research, which is very useful.	Quảng Ninh
	Some lectures were not updated, although the official documents and national treatment guides are changed frequently.	Thái Nguyên
	The current explanation in the links was short and insufficient; there should be more information so we wouldn’t have to access other sites.	Thái Nguyên
Subject matter expertise	Unlike those of you who have been in this program for one year and have experience and knowledge, I am totally new to this field and I need to search for information.	Quảng Ninh
	People who are new to this information will be more motivated to study. I have already been trained about this before so I don’t actively participate in the courses, although I think they are useful for me.	Quảng Ninh
Technology literacy or accessibility	The password is hard to remember. Sometimes I have to type it from the beginning.	Quảng Ninh
	My limitation would be my bad eyesight. I would only read the links if I was on the computer.	Thái Nguyên
	It may take 1 or 2 weeks for us to remember the phone number [text message] of the program.	Hải Phòng
Motivation	I like it best when having group discussion, sending the answer, and then receiving “Congratulations” on giving the right answer.	Quảng Ninh
	If studying time is taken into account to decide whether or not one could receive a certificate, I will have a purpose and be more motivated to study.	Quảng Ninh
	When I have answered too many quizzes and my score is low compared to the group average, I will read the daily readings to gain more knowledge.	Hải Phòng

One participant said, “If I need the knowledge at that moment, I will be more motivated to study” (participant in Quảng Ninh), but another commented that, “I would spend more time studying work-related topics, I researched more and often got high score; other than that I would skip [questions]” (participant in Thái Nguyên). Similarly, whether they viewed mCME as convenient affected their study behaviors. One participant said that:

The most beneficial thing for us working at medical centers I think is that it can be studied anywhere, without having to attend classes, and it is suitable for those who live far away from the training center.

participant in Thái Nguyên

A counterpart said:

Actually, there are very few people who have time to participate in the courses at home, and studying when working often gets interrupted. Sometimes it is possible to read the entire lectures but sometimes I have to turn it off after having read for only 1 or 2 minutes.

participant in Quảng Ninh

Ultimately, these modifying factors boil down to the individual participant. One health professional from Thái Nguyên succinctly summarized, noting that “Compared to direct training, I think online learning is very good, with the condition that the learner has the will to learn.”

### Impacts of the Intervention on Self-Study and Medical Knowledge

The mCME V.2.0 intervention significantly increased self-study behaviors, leading to improved HIV medical knowledge and perceived skills, and high levels of job satisfaction [18]. In particular, it was the unique combination of these multiple components of the intervention—the cues to action (daily quizzes and individual or peer feedback) and links to self-study resources (daily readings and HMU courses)—that was appealing to participants. When asked which of the components was most useful, nearly half chose all 3, rather than selecting only one.

#### Impact on HIV Knowledge

In mCME V.2.0, intervention participants accessed HMU courses and daily readings significantly more than the control group did, and response rates to the daily quizzes remained high throughout the trial. This resulted in a significant difference in examination scores between intervention and control participants [18]. Of the 48 intervention participants, 83% (n=40) felt that they were better prepared to care for patients with HIV in their communities, compared with 67% (n=32) at baseline. In the FGDs, participants reported similarly that the intervention improved their knowledge of HIV. One participant said that, “At first I didn’t know much, but after that I still needed to read more materials and use the internet to improve my knowledge” (participant in Thái Nguyên). Another noted that, “The benefit is that [the quizzes] motivated me to search for information in order to answer questions in the field that I do not have much knowledge about” (participant in Hải Phòng).

#### Impact on HIV Feelings of Self-Efficacy

Our intervention did not evaluate clinical skills, but we did ask participants to comment on their own abilities pertaining to HIV care. Figure 2 illustrates additional self-assessment data of HIV knowledge and self-efficacy. Of particular note, 85% (41/48) of intervention participants reported that they either agreed or strongly agreed with the statement “I feel confident providing clinically accurate HIV diagnoses and counseling to patients,” and 94% (45/48) agreed or strongly agreed with the statement “I feel confident in my ability to educate patients about HIV prevention” (Figure 2). However, none of these self-assessments different significantly from the control group, with the exception of the statement “I feel confident in my ability to appropriately identify patients who require methadone treatment.” One FGD participant summed up these views, saying that, “The most important benefit is that [the courses] increase our knowledge and help us become more confident” (participant in Hải Phòng).

## Discussion

### Principal Findings

Data from this randomized controlled trial among HIV clinicians in Vietnam may suggest that a mobile CME intervention is both effective at improving medical knowledge and self-study behaviors, and acceptable by trial participants. Participants reported in our postintervention survey and FGDs that they liked the 3 components of the intervention: the daily quizzes, the daily readings, and the HMU online courses. FGD participants noted that the daily quizzes encouraged them to seek out answers and learn on a daily basis, but also commented that clarity of the messaging and the timing of the messages were sometimes an issue. HMU course utilization rates were significantly higher in the intervention group, but participants noted that the quality, format, and loading time of the HMU courses precluded additional use. The HMU courses themselves were unpopular; participants’ use of this resource was low, and public health officials should address feedback on these resources when developing new content for their platforms. The daily readings were not well used during the intervention; some participants found them to be too short to provide sufficient information. However, in the context of mCME, many noted that the readings were helpful in that they provided immediate and accurate answers to the daily quiz questions. We conclude that the intervention was acceptable, was convenient, and helped improve health professionals’ knowledge of HIV treatment and care in Vietnam within this population of HIV providers.

Although we showed the intervention to improve medical knowledge, disaggregating the 3 components of the intervention to understand which was the most influential remains a challenge. A medical degree, or even CME, given over SMS text messaging is not sufficient, and content without practical training is not sufficient for health professionals. We posit that it was not the SMS text messages, but rather the stimulus that they provided to seek out answers and improve their self-study via digital and interprofessional resources, that was responsible for the improvement in medical knowledge. The survey and FGD data presented in this analysis supports this hypothesis, and we also explored this further in a separate quantitative analysis of the factors that led participants to engage in self-study behaviors [19].

Many factors interrelated to facilitate or prevent self-study in this population, as outlined in Figure 1 and Table 2. For example, the convenience of mCME, combined with the feeling that the topic area was professionally relevant, could have motivated a participant to study. Inversely, lack of free time coupled with the feeling that the participant was already an expert in the subject matter could have discourage one from doing so. These factors, considered both individually and with others, may have been important influencers in a participant’s decision to engage in lateral learning during the intervention period.

When considering the variables that influence a clinician’s decision to seek out in-depth information, we must consider these aforementioned variables, as well as the larger framework of the HBM. This model posits that an individual’s perceived severity of disease and perceived susceptibility to disease, along with cues to action and moderating variables, all contribute to the decision-making process of whether to engage in a health behavior [20,21]. Applied to our research setting, the HBM can be used to consider the different factors that influence a clinician’s decision to engage in lateral learning (Figure 1). Following this framework, intervention participants chose to both engage and not engage in lateral learning, based on the personal value placed on the modifying factors and their perspectives on the need and importance of CME. In this research study, the intervention group had a larger change in performance between baseline and endline examinations than did controls, who did not have any of the cues to action listed in this model [18]. Past research is consistent with the findings of this analysis, which postulate that, ultimately, medical professionals must find the internal motivation to learn and must view learning both as beneficial to their practice and as something they are capable of doing [22,23].

Multiple theories have been put forward in the field of CME to enhance learning, and these theories need to be applied when developing distance CME programs in the future [24]. This research intervention attempted to answer the question of whether CME might be delivered at a distance, and our conclusion is that this is a viable method, provided that we employ behavior change learning models rather than purely educational ones. Our research framework considered the HBM but adapted it to a pedagogical setting, providing public health professionals with the successful structure that could be repeated and adapted to future iterations of this program at a national scale. Additional quantitative research is recommended in this area to support generalizability to other settings and populations, and to further explore the modifying factors that ultimately influence access, use, and learning.

### Limitations

Our study had several limitations. With only 106 health professionals enrolled, our sample size was relatively small, which suggests that this research should be repeated at a larger scale to further inform precise impact measures. However, it is important to note that we selected participants via rigorous sampling and randomization methods, and we were able to capture nearly all eligible participants across the 3 provinces. Additionally, participants in qualitative research are always subjected to social desirability bias, which could mean that the statements made may not accurately reflect their actions so much as their desire to appear to be good students. We mitigated this by triangulating our quantitative and qualitative results and including opinions of only those who ever accessed the components of the intervention when reporting on their views of the intervention. And lastly, our research measured HIV medical knowledge, which is related to but not the same as clinical practice. Future research should explore the modifying factors that affect learning and measure whether CME programs improve clinical diagnoses, treatment, and general performance.

### Conclusion

In this randomized controlled trial, we have proven that the intervention (1) improved self-study behaviors, (2) improved medical knowledge, and (3) was acceptable to the target population. This mixed-methods analysis demonstrated that the intervention was easy to use, convenient, and relevant to the participants’ work. Factors such as topic relevance, subject matter expertise, and availability of high-quality resources affected participants’ decisions to access additional resources. If public health officials choose to scale up this platform to the national level, future programs may consider the lateral learning framework as part of a successful strategy to engage in higher learning and truly improve medical knowledge. mCME was well liked, inexpensive, and cost effective, and thus merits consideration for nationwide scale-up. If implemented, mCME has the potential to improve the medical knowledge of health professionals across multiple disciplines.

## Appendix

Multimedia Appendix 1 Study design of mCME V.2.0 (November 2016 to May 2017).

Multimedia Appendix 2 Impact of timing of daily quiz on response rate, correct answer rate, and use of daily readings.

## Abbreviations

CME: continuing medical education
FGD: focus group discussion
HBM: health belief model
HMU: Hanoi Medical University
mCME: Mobile Continuing Medical Education Project
mHealth: mobile health
SMS: short message service
