# Supplementary figures and images for: Motivating HIV Providers in Vietnam to Learn: A Mixed-Methods Analysis of a Mobile Health Continuing Medical Education Intervention
Source: JMIR Med Educ. 2019 Apr 18;5(1):e12058. doi: 10.2196/12058 (PMC6495296; doi:10.2196/12058)

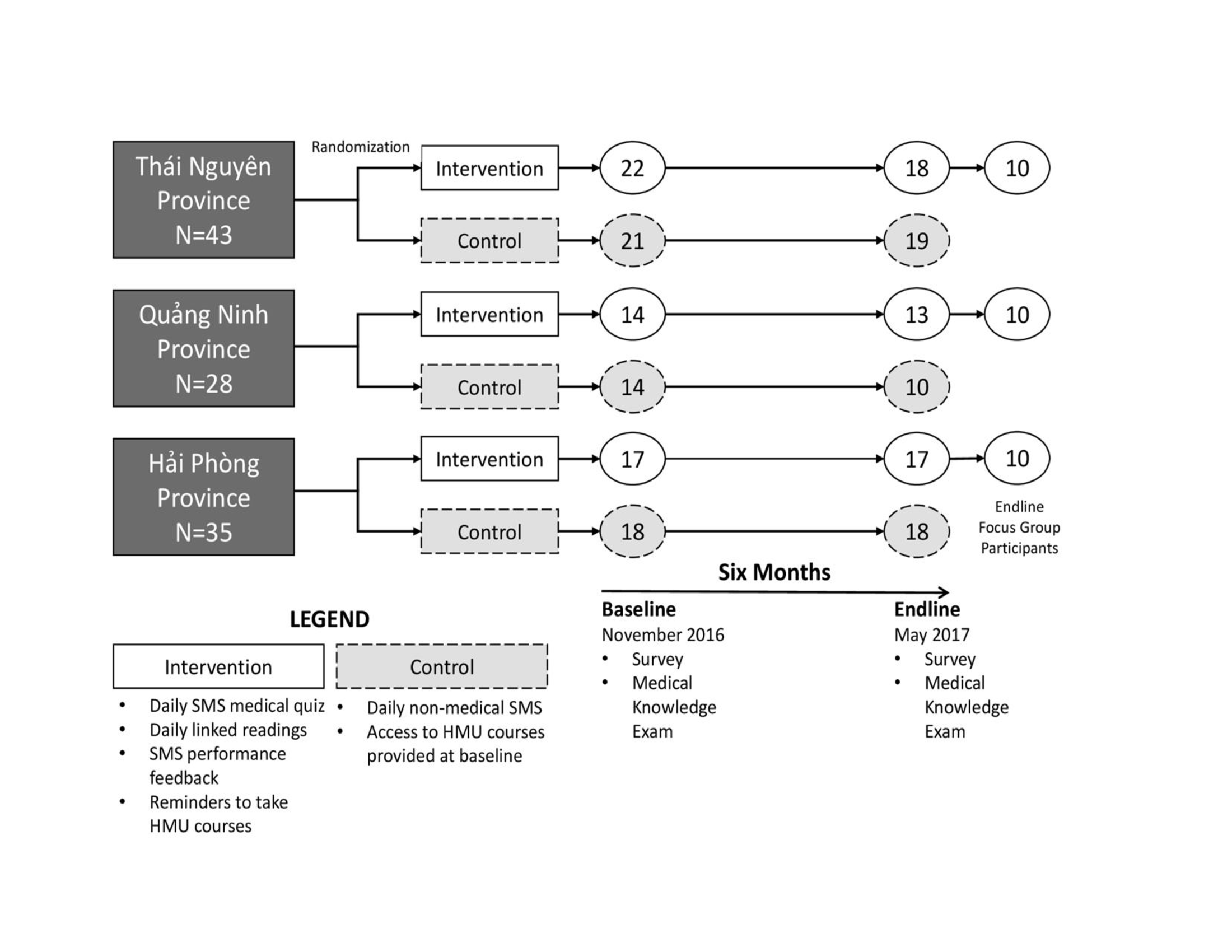

Supplement: Multimedia Appendix 1 [file mededu_v5i1e12058_app1.png]

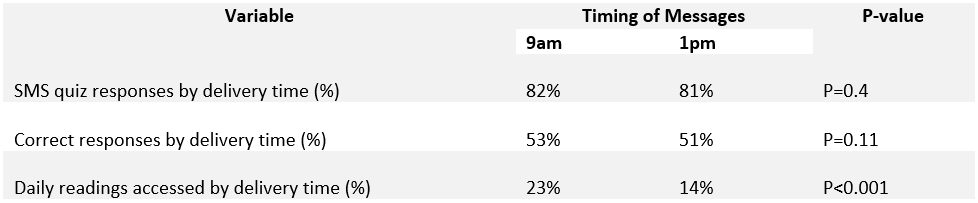

Supplement: Multimedia Appendix 2 [file mededu_v5i1e12058_app2.PNG]
